# Replicated evidence for an accelerated rate of whole-body aging in schizophrenia

**DOI:** 10.1017/S003329172610333X

**Published:** 2026-02-09

**Authors:** Ethan T. Whitman, Roberta Passiatore, Annchen R. Knodt, Giulio Pergola, Linda A. Antonucci, Alessandro Bertolino, Giuseppe Blasi, Enrico D’Ambrosio, Maxwell L. Elliott, Gianluca C. Kikidis, Annalisa Lella, Antonella Lupo, Alessandra Raio, Antonio Rampino, Nicola Sambuco, Pierluigi Selvaggi, Daniel R. Weinberger, Terrie E. Moffitt, Avshalom Caspi, Ahmad R. Hariri

**Affiliations:** 1 Department of Psychology and Neuroscience, Duke University, Durham, NC, USA; 2 Lieber Institute for Brain Development, Johns Hopkins Medical Campus, Baltimore, MD, USA; 3 Department of Translational Biomedicine and Neuroscience (DiBraiN), University of Bari Aldo Moro, Bari, Italy; 4 Department of Psychiatry and Behavioral Sciences, Johns Hopkins School of Medicine, Baltimore, MD, USA; 5 Department of Genetic Medicine, Johns Hopkins School of Medicine, Baltimore, MD, USA; 6 Azienda Ospedaliero-Universitaria Consorziale Policlinico, Bari, Italy; 7 Department of Psychology, Center for Brain Science, Harvard University, Cambridge, MA, USA; 8 Department of Psychology, University of Minnesota, Minneapolis, MN, USA; 9 Department of Neurology, Johns Hopkins University School of Medicine, Baltimore, MD, USA; 10 King’s College London, Social, Genetic, and Developmental Psychiatry Centre, Institute of Psychiatry, Psychology, and Neuroscience, London, UK; 11 PROMENTA, Department of Psychology, University of Oslo, Oslo, Norway; 12 Department of Psychiatry and Behavioral Sciences, Duke University, Durham, NC, USA

**Keywords:** aging, MRI, neuroimaging, schizophrenia, psychosis, clinical high risk for psychosis

## Abstract

**Background:**

People with schizophrenia develop more chronic diseases at a younger age and die younger than people in the general population. It has been hypothesized that this excess morbidity and mortality could be partially due to accelerated aging in schizophrenia. If true, this would motivate the development of ‘gero-protective’ interventions to reduce chronic disease burden in schizophrenia. However, it has been difficult to test this hypothesis, in part, due to the limited ability to measure aging in samples of people with schizophrenia.

**Methods:**

We utilized a novel neuroimaging biomarker of the longitudinal pace of aging, DunedinPACNI, to test for accelerated whole-body aging in schizophrenia across four neuroimaging datasets (total *N* = 2,096, 48% female) accessed through the Lieber Institute for Brain Development, the University of Bari Aldo Moro, and the North American Prodrome Longitudinal Study – 3.

**Results:**

We found consistent evidence of faster DunedinPACNI in schizophrenia compared with controls. In contrast, youth at clinical-high risk for psychosis did not have faster DunedinPACNI compared to controls. Unaffected siblings of patients also did not have faster DunedinPACNI than controls. Faster DunedinPACNI in schizophrenia was not explained by tobacco smoking or antipsychotic medication use.

**Conclusions:**

The results support the hypothesis that schizophrenia is accompanied by accelerated aging. Results were inconsistent with some of the most obvious explanations for accelerated aging in schizophrenia (familial risk, smoking, and iatrogenic medication effects). Research should aim to uncover why people who have schizophrenia age rapidly, as well as the utility of early disease-risk monitoring and anti-aging interventions in schizophrenia.

## Introduction

Schizophrenia is associated with increased incidence of multiple chronic age-related diseases such as type II diabetes, chronic obstructive pulmonary disease, heart disease, and dementia (Correll et al., 2017; Mitchell et al., 2013; Momen et al., 2020; Richmond-Rakerd et al., 2022; Rossom et al., 2022; Stroup et al., 2021; Suetani et al., 2021). Patients with schizophrenia experience these diseases at a younger age than the general population, contributing to decreased life expectancy (Hjorthøj, Stürup, McGrath, & Nordentoft, 2017). These observations have led to the hypothesis that schizophrenia is associated with accelerated aging (Jeste, Wolkowitz, & Palmer, 2011; Kirkpatrick et al., 2008; Kochunov & Hong, 2024; Nguyen, Eyler, & Jeste, 2018). However, research on accelerated aging in schizophrenia has been hampered by the lack of consensus tools for assessing how fast people age (Cohen et al., 2020).

There are multiple different approaches used to measure aging. One popular method is called ‘age deviation.’ In this method, researchers use biological data (e.g. neuroimaging, DNA methylation, proteomics) to train machine-learning models that can estimate chronological age (Clausen et al., 2022; Hannum et al., 2013; Horvath, 2013; Oh et al., 2023; Tian et al., 2023). Next, researchers apply these models to unseen people and identify the deviation between the estimated age and actual age. If a person’s estimated age is greater than their actual age, this is interpreted as accelerated aging (Clausen et al., 2022; Hannum et al., 2013; Horvath, 2013; Oh et al., 2023; Tian et al., 2023). While age-deviation measures are often correlated with disease (Franke & Gaser, 2019; Oh et al., 2023; Tian et al., 2023), these measures equate model error (e.g., historical differences in environmental exposures, survivor bias, disease effects, measurement bias) with a person’s true rate of aging-related decline (Butler et al., 2021; Sluiskes et al., 2024). As a result of this limitation, we recently developed a longitudinal approach to measuring aging in the Dunedin Study, a population-representative birth cohort followed from birth until age 45. We first measured longitudinal change of 19 biomarkers indexing several organ systems from ages 26–45 years to create a summary measure of whole-body aging: the Pace of Aging (Elliott et al., 2021). This measure operationalizes aging as a correlated gradual decline across organ systems and reflects differences among individuals in their rate of biological aging by midlife. Next, using DNA methylation data from whole blood at age 45, we developed an algorithm that accurately estimates the longitudinal Pace of Aging. This measure is called the Dunedin Pace of Aging Calculated from the Epigenome: DunedinPACE (Belsky et al., 2022). Of note, prior studies in schizophrenia using epigenetic ‘age deviation’ measures found inconsistent and often null results (Chrusciel et al., 2022; Higgins-Chen et al., 2020; Kowalec et al., 2019; Li et al., 2023; Ori et al., 2019; Wu, Ye, Wang, & Zhao, 2021), whereas a recent study using DunedinPACE identified consistent patterns of accelerated aging in schizophrenia (Caspi et al., 2024).

So far, studies of schizophrenia using neuroimaging-based age biomarkers have utilized exclusively ‘age deviation’ style measures – often referred to as ‘brain age gap’ (Biondo et al., 2022; Cole et al., 2018). Large studies show that schizophrenia patients have a larger brain age gap than healthy controls (Constantinides et al., 2023; Kaufmann et al., 2019; Tønnesen et al., 2020). Following the model of DunedinPACE, we recently developed a neuroimaging-based measure that accurately estimates the longitudinal Pace of Aging phenotype from a single T1-weighted structural MRI of the brain. This measure is called the Dunedin Pace of Aging Calculated from NeuroImaging: DunedinPACNI. This method exploits bidirectional influences of aging of the body and the health of the brain (Tian et al., 2023) to accurately estimate whole-body aging according to brain structure. Specifically, faster DunedinPACNI is reflected in a widely distributed pattern of thinner cerebral cortex, smaller gray matter volume, lower gray-white matter signal intensity ratio, and larger ventricular volume. Faster DunedinPACNI predicts cognitive decline, dementia conversion, incident disease, and mortality (Whitman et al., 2025). Of note, DunedinPACNI is only modestly correlated with brain age gap and appears more sensitive to clinical outcomes compared to brain age gap (Whitman et al., 2025). It is not known whether DunedinPACNI would identify patterns of accelerated aging in schizophrenia, nor how these patterns may be related to brain age gap.

Here, we test the link between schizophrenia and accelerated aging measured via DunedinPACNI. First, we estimated DunedinPACNI in four datasets totaling 2,096 individuals, allowing us to establish whether there is replicable evidence of accelerated aging in schizophrenia. Second, three of the four datasets included unaffected siblings of schizophrenia patients, allowing us to compare affected and unaffected siblings. This comparison provided a way to rule out the possibility that shared familial risk factors account for the link between schizophrenia and accelerated aging. Third, one of the four datasets included youth at clinical high-risk for psychosis who show subthreshold clinical symptoms but do not meet criteria for schizophrenia. This allowed us to test whether evidence of accelerated aging is apparent among people with subthreshold signs of psychosis who have not converted. Fourth, we conducted sensitivity analyses to assess whether faster DunedinPACNI is driven by higher rates of tobacco smoking in schizophrenia (Winterer, 2010) or by antipsychotic medication, which may have deleterious physical-health side effects (Jonas, Abi-Dargham, & Kotov, 2021; Taipale et al., 2020). Finally, we tested whether DunedinPACNI is associated with accelerated aging in schizophrenia over and above brain age gap.

## Methods

Analyses were conducted on data provided through the Lieber Institute for Brain Development (LIBD), the University of Bari Aldo Moro (UNIBA), and the North American Prodrome Longitudinal Study–3 (NAPLS-3). Details about each dataset are described elsewhere (Addington et al., 2022; Antonucci et al., 2016; Egan et al., 2001). Sample demographics are provided in Table 1. All analyses and code were checked for accuracy by an independent analyst. The authors assert that all procedures contributing to this work comply with the ethical standards of the relevant national and institutional committees on human experimentation and with the Helsinki Declaration of 1975, as revised in 2008.

**Table 1.** Sample demographic information

Dataset	*N*	Mean age	SD age	Female (%)
**LIBD**	**416**	**30.88**	**9.67**	**51%**
SCZ	112	30.81	9.85	31%
SIB	46	31.11	10.34	54%
NC	258	30.87	9.50	59%
**UNIBA–1**	**694**	**28.54**	**8.73**	**50%**
SCZ	113	33.19	8.97	29%
SIB	59	36.59	8.99	54%
NC	522	26.62	7.71	54%
**UNIBA–2**	**325**	**28.58**	**8.99**	**51%**
SCZ	66	31.48	9.83	26%
SIB	11	43.09	8.01	64%
NC	248	27.16	8.02	57%
**NAPLS–3**	**661**	**19.39**	**4.10**	**44%**
NC	90	19.69	4.41	49%
CHR	571	19.32	4.03	44%

Abbreviations: CHR: clinical high-risk for psychosis; HC: healthy control; LIBD: Lieber Institute for Brain Development; NAPLS-3: North American Prodrome Longitudinal Study - 3; SCZ: schizophrenia; SD: standard deviation; SIB: sibling; UNIBA: University of Bari.

### Datasets

#### LIBD

All patients met DSM-IV criteria for schizophrenia or related diagnoses, including schizoaffective disorder, psychosis (not otherwise specified), and schizoid, paranoid, and schizotypal personality disorders. Patients with a history of other psychiatric illnesses were excluded. A minority of unaffected first-degree siblings had a past lifetime history of a non-psychotic mental illness and/or substance abuse and/or dependence (39.7%), but none met criteria at the time of evaluation. Healthy controls had no lifetime history of psychiatric disorders. Total lifetime antipsychotic exposure was measured using chlorpromazine equivalents (Gardner et al., 2010). Smoking was measured as self-reported packs of cigarettes smoked per day. T1-weighted images were acquired on a 3T MRI scanner and processed using FreeSurfer version 6.0. Data were excluded if images failed FreeSurfer QC or if segmentation failed visual inspection. MRI details have been reported previously (Lella et al., 2025).

#### UNIBA

All patients met DSM-IV criteria for schizophrenia, while unaffected first-degree siblings of patients and healthy controls were included if they had no lifetime history of psychiatric disorders. Total lifetime antipsychotic exposure was measured using chlorpromazine equivalents (Gardner et al., 2010). Smoking was measured as self-reported packs of cigarettes smoked per day. T1-weighted images were acquired on two 3T MRI scanners. To mitigate potential scanner effects, we treated data from each scanner as a unique dataset. We refer to these as UNIBA-1 and UNIBA-2. Data from UNIBA-1 and UNIBA-2 were processed using FreeSurfer version 7.2. Data were excluded if images failed FreeSurfer QC or if segmentation failed visual inspection. MRI details have been reported previously (Fazio et al., 2023).

#### NAPLS-3

All participants categorized as clinical high-risk met the Criteria of Psychosis Risk Syndromes, based on the Structured Interview for Psychosis-Risk Syndromes (McGlashan, Walsh, & Woods, 2010). T1-weighted images at all timepoints were acquired using 3T scanners and processed using longitudinal FreeSurfer version 6.0. Scans were excluded if they failed FreeSurfer QC or if segmentation failed visual inspection. MRI details have been reported previously (Addington et al., 2022).

### Statistical analyses

#### DunedinPACNI

We derived DunedinPACNI from processed T1-weighted images using the publicly available algorithm (https://github.com/etw11/DunedinPACNI). Briefly, DunedinPACNI was derived using elastic net regression applied to 315 structural brain phenotypes, including regional cortical thickness, surface area, gray matter volume, gray-white matter signal intensity ratio, subcortical gray matter volumes, ventricular volumes, and bilateral volume of white matter hypointensities. Within each dataset, DunedinPACNI values were standardized to mean = 0, standard deviation = 1. DunedinPACNI details have been reported previously (Whitman et al., 2025).

#### Association with diagnosis

In three datasets, we tested for group differences in DunedinPACNI between (i) patients with schizophrenia versus healthy controls, (ii) patients with schizophrenia versus unaffected siblings, and (iii) unaffected siblings versus healthy controls. We also tested for faster DunedinPACNI in patients compared to their specific unaffected siblings using nested random effects for dataset and family. Finally, we tested for group differences in DunedinPACNI between youth at clinical high-risk for psychosis and healthy controls. We included a random effect for the individual to control for within-individual nesting among clinical high-risk participants who had more than one scan. All analyses controlled for age and sex.

#### Sensitivity analyses

We tested the association between schizophrenia and DunedinPACNI while including packs of cigarettes smoked per day and lifetime chlorpromazine equivalents, respectively, as covariates in LIBD, UNIBA-1, and UNIBA-2. Because many participants were missing these measures, we pooled data across datasets to perform a mega-analysis while covarying for dataset (see Supplemental Materials S1–S2). Although all analyses controlled for chronological age, we also undertook two sensitivity analyses to test for age effects. First, due to minor differences in chronological age between cases and controls in UNIBA-1 and UNIBA-2, we repeated case–control analyses in these cohorts while analyzing an age-matched subset of healthy control participants. These subsets were generated by excluding healthy controls younger than 27 years old in UNIBA-1 and 24 years old in UNIBA-2. Age distributions of healthy control participants in the main and sensitivity analyses of all datasets are presented in Supplementary Figure S1. Second, we stratified the LIBD, UNIBA-1, and UNIBA-2 schizophrenia patients and healthy control subjects into tertiles based on chronological age. We then repeated case–control tests while controlling for age and sex within each tertile. Finally, we sought to test whether the effect sizes in LIBD were sensitive to the broader set of psychotic diagnoses included in that dataset. To do this, we tested case–control differences in LIBD only among patients diagnosed with schizophrenia, thereby excluding patients with diagnoses of schizoaffective disorder, psychosis (not otherwise specified), and schizoid, paranoid, and schizotypal personality disorders.

#### Comparison with brain age gap

We benchmarked DunedinPACNI findings against brain age gap, a first-generation MRI-based ‘age deviation’ biomarker. We used brainageR (Biondo et al., 2022) to estimate chronological age with the same T1-weighted images used to derive DunedinPACNI. We subtracted chronological age from MRI-predicted age to yield a brain age gap score for each participant and repeated all analyses with this score. Next, we tested whether DunedinPACNI and brain age gap were independently associated with schizophrenia or clinical high-risk for psychosis by including DunedinPACNI and brain age gap in the same model. Finally, we meta-analyzed DunedinPACNI and brain age gap effects using the *meta* R package (Schwarzer, Carpenter, & Rucker, 2015).

## Results

### Schizophrenia is associated with faster DunedinPACNI

**Figure 1.**
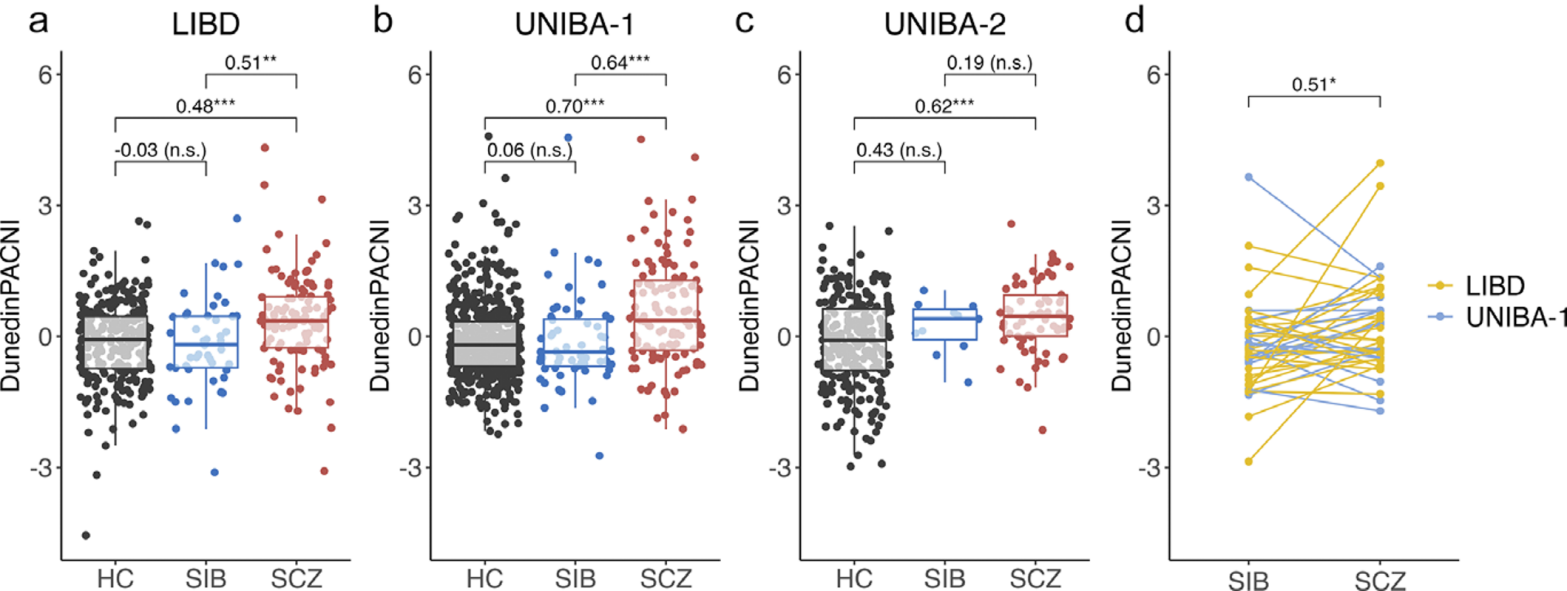
Group differences in the pace of aging as measured by DunedinPACNI. (a)–(c) Boxplots of DunedinPACNI in patients with schizophrenia, unaffected first-degree siblings, and healthy controls in the LIBD (a), UNIBA-1 (b), and UNIBA-2 (c) datasets. Brackets represent standardized group differences while controlling for age and sex. Within each dataset, DunedinPACNI values were standardized to mean = 0, standard deviation = 1. (d) Within-family comparison of DunedinPACNI in the LIBD and UNIBA-1 datasets. Each point represents a unique individual and each line connects a pair of siblings. Yellow points represent participants from LIBD and blue points represent participants from UNIBA-1. Bracket represents group differences while controlling for age and sex with nested random effects for dataset and family. ***p < 0.001; **p < 0.01; *p < 0.05. Abbreviations: HC: healthy control; LIBD: Lieber Institute for Brain Development; n.s.: not statistically significant; SCZ: schizophrenia; SIB: sibling; UNIBA: University of Bari.

We measured each participant’s pace of aging by calculating DunedinPACNI from T1-weighted structural brain scans. In all three adult schizophrenia datasets, patients had faster DunedinPACNI compared to healthy controls (LIBD: *β* = 0.48, *p* < 0.001, 95%CI: [0.27–0.69]; UNIBA-1: *β* = 0.70, *p* < 0.001, 95%CI: [0.50–0.90]; UNIBA-2: *β* = 0.62, *p* < 0.001, 95%CI: [0.36–0.89]; Figure 1a–c, Supplementary Table S1). In general, patients with schizophrenia also had faster DunedinPACNI compared to unaffected siblings (LIBD: *β* = 0.51, *p* = 0.001, 95%CI: [0.20–0.83]; UNIBA-1: *β* = 0.64, *p* < 0.001, 95%CI: [0.34–0.94]; UNIBA-2: *β* = 0.19, *p* = 0.53, 95%CI: [−0.42–0.81]; Figure 1a–c, Supplementary Table S1). We did not observe any significant differences in DunedinPACNI between unaffected siblings and healthy controls (LIBD: *β* = −0.03, *p* = 0.82, 95%CI: [−0.32–0.25]; UNIBA-1: *β* = 0.06, *p* = 0.67, 95%CI: [−0.21–0.32]; UNIBA-2: *β* = 0.43, *p* = 0.16, 95%CI: [−0.16–1.02], Figure 1a–c, Supplementary Table S1).

We also compared sibling pairs within the same family. To do so, we compared 36 patients with schizophrenia with their 42 unaffected siblings, using all available pairs of siblings who were enrolled in LIBD and UNIBA-1 (UNIBA-2 was not included as there was only one sibling pair with MRI data). Schizophrenia patients had faster DunedinPACNI compared to their siblings (*β* = 0.51, 95%CI: [0.04–0.99], *p* = 0.04, Figure 1d).

### Clinical high-risk for psychosis is not associated with DunedinPACNI

**Figure 2.**
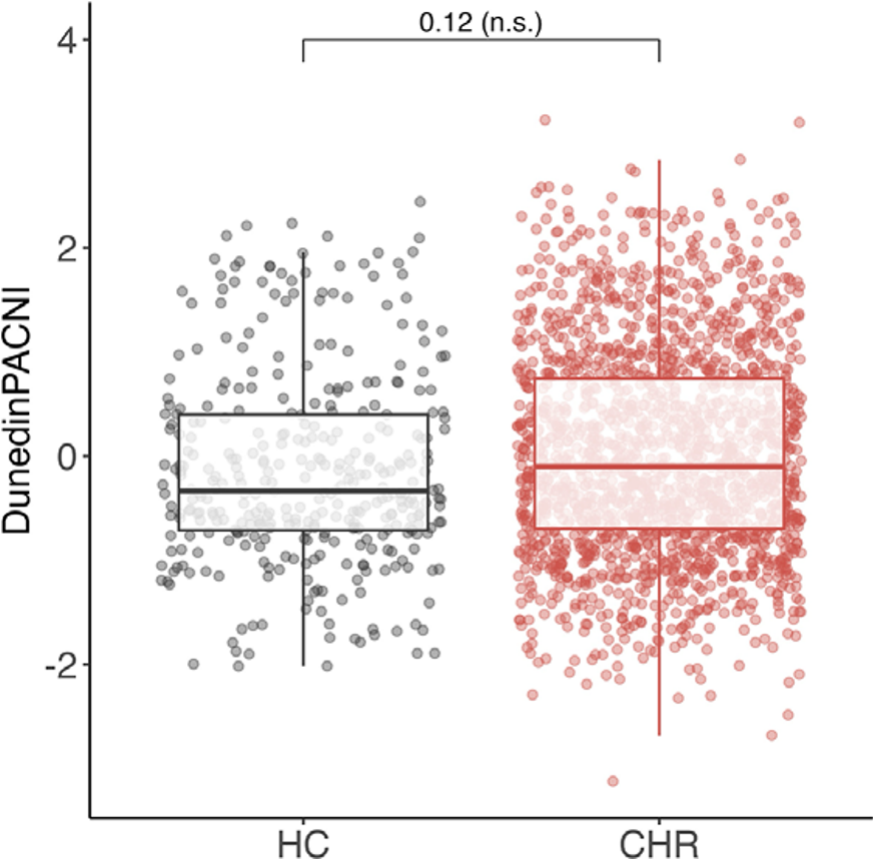
DunedinPACNI in youth at clinical high-risk for psychosis. Boxplot of DunedinPACNI in youth at clinical high-risk for psychosis and healthy controls from the NAPLS-3 dataset. Bracket represents standardized group differences while controlling for age and sex and using a random effect for individual to account for multiple observations. Abbreviations: CHR: clinical high-risk for psychosis; HC: healthy control; n.s.: not statistically significant.

We measured each participant’s pace of aging in NAPLS-3 by calculating DunedinPACNI from T1-weighted structural brain scans. In contrast to the three adult samples of patients with schizophrenia, youth at clinical high-risk for psychosis did not have significantly faster DunedinPACNI compared to healthy controls (*β* = 0.12, *p* = 0.28, 95%CI: [−0.10–0.37]; Figure 2, Supplementary Table S1).

### Sensitivity analyses

We tested whether tobacco smoking accounted for accelerated DunedinPACNI in patients with schizophrenia. Because only 745 out of 1,435 adult participants across datasets had available smoking data, we pooled data across LIBD, UNIBA-1, and UNIBA-2 (see Supplementary Material S1). Patients with schizophrenia smoked more tobacco than controls (*β* = 0.93, *p* < 0.001, 95%CI: [0.73–1.12]), but there was no significant association between smoking and DunedinPACNI (*β* = 0.04, *p* = 0.21, 95%CI: [−0.02–0.10]; Supplementary Figure S2). Moreover, we continued to observe faster DunedinPACNI in schizophrenia compared to healthy controls while covarying for smoking (*β* = 0.32, *p* = 0.001, 95%CI: [0.13–0.51]).

We conducted parallel sensitivity analyses to test whether antipsychotic medication accounted for accelerated aging in patients with schizophrenia. We again pooled data to mega-analyze the 198 schizophrenia patients with available medication data out of the 291 total patients from LIBD, UNIBA-1, and UNIBA-2 (see Supplementary Material S2). We did not observe significant associations between total chlorpromazine equivalents and DunedinPACNI (*β* = 0.08, *p* = 0.26, 95%CI: [−0.06–0.23]; Supplementary Figure S3), and continued to observe faster DunedinPACNI scores in schizophrenia patients compared to healthy controls while covarying for total chlorpromazine equivalents (*β* = 0.44, *p* = 0.002, 95%CI: [0.16–0.71]).

Furthermore, we tested whether differences in DunedinPACNI in UNIBA-1 and UNIBA-2 were driven by small differences in chronological age between schizophrenia patients and controls (Supplementary Figure S1). To do so, we generated age-matched subsets of healthy controls in UNIBA-1 and UNIBA-2 (age-matched control subsets in UNIBA-1: *N* = 180, mean age = 34.7 (SD age = 8.0), female = 41%; in UNIBA-2: *N* = 156, mean age = 30.6 (SD age = 8.4), female = 51%; Supplementary Figure S1). We continued to observe faster DunedinPACNI scores in schizophrenia patients in UNIBA-1 and UNIBA-2 compared to age-matched subsets of healthy controls (UNIBA-1: *β* = 0.69, *p* < 0.001, 95%CI: [0.43–0.95]; UNIBA-2: *β* = 0.64, *p* < 0.001, 95%CI: [0.37–0.90]). This sensitivity analysis was not necessary in LIBD or NAPLS-3 because these datasets had no differences in age between cases and controls (Supplementary Figure S1). In addition, we observed stepwise increases in effect size when stratifying case–control tests by age (Supplementary Figure S4, Supplementary Table S2), suggesting that the pattern of faster DunedinPACNI in schizophrenia is more pronounced among older participants. Finally, we observed faster DunedinPACNI scores in cases compared to controls in LIBD while only analyzing patients with diagnosed schizophrenia (*N* = 96; *β* = 0.51, *p* < 0.001, 95%CI: [0.29–0.73]). This sensitivity analysis was not necessary in UNIBA-1 or UNIBA-2, because all patients in these datasets had a diagnosis of schizophrenia.

### Comparing biological aging measured via DunedinPACNI and brain age gap

**Figure 3.**
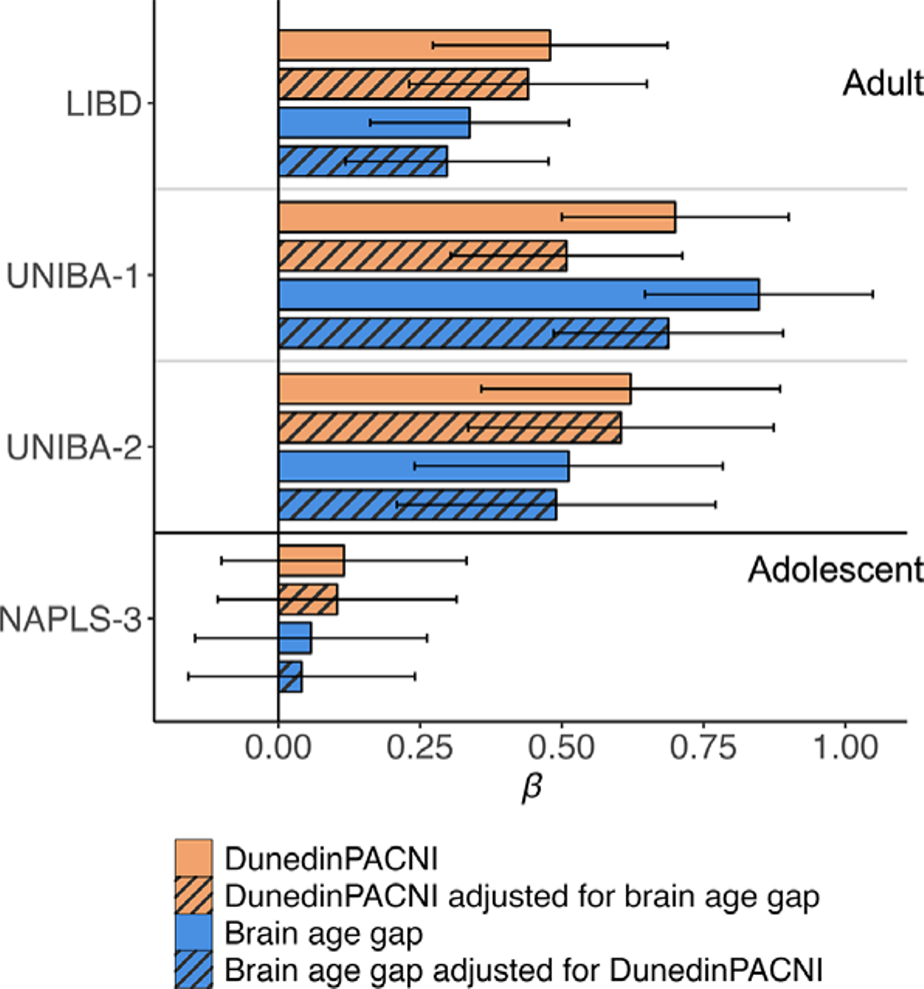
Comparison of DunedinPACNI and brain age gap. Bar plot of standardized group differences in DunedinPACNI and brain age gap between patients with schizophrenia and healthy controls (LIBD, UNIBA-1, UNIBA-2), as well as youth at clinical high-risk for psychosis and healthy controls (NAPLS-3). Orange bars show effect sizes for DunedinPACNI, blue bars show effect sizes for the brain age gap. Hashed bars show effect sizes for each measure (e.g. DunedinPACNI or brain age gap) while controlling for the other. Error bars represent 95% confidence intervals while controlling for age and sex. A random effect for individual was included for NAPLS-3 models to account for multiple observations. Abbreviations: CHR: clinical high-risk for psychosis; HC: healthy control; LIBD: Lieber Institute for Brain Development; NAPLS-3: North American Prodrome Longitudinal Study - 3; SCZ: schizophrenia, UNIBA: University of Bari.

We computed brain age gap in all four datasets. Consistent with prior work (Whitman et al., 2025), we observed weak correlations between DunedinPACNI and brain age gap across datasets, suggesting little overlap in variance captured by each measure (LIBD: *r* = −0.12, *p* = 0.013; UNIBA-1: *r* = 0.24, *p* < 0.001; UNIBA-2: *r* = −0.03, *p* = 0.96; NAPLS-3: *r* = 0.20, *p* < 0.001). Schizophrenia was also associated with a higher brain age gap in all adult datasets, though clinical high-risk status was not associated with brain age gap in youth (Figure 3, Supplementary Table S3). Brain age gap differences between pairs of patients and their unaffected siblings in LIBD and UNIBA-1 were smaller than those for DunedinPACNI (brain age gap: *β* = 0.20, 95%CI: [−0.20–0.61, *p* = 0.11 vs. DunedinPACNI: (*β* = 0.51, 95%CI: [0.04–0.99], *p* = 0.04). Crucially, when we included both DunedinPACNI and brain age gap in a single model, we observed only minor attenuations in association with diagnosis (Figure 3, Supplementary Table S3).

The meta-analysis of patients with schizophrenia and healthy controls across all adult samples (LIBD, UNIBA-1, UNIBA-2) revealed comparable effect sizes for DunedinPACNI (random effects model: *β* = 0.60, *p* < 0.001, 95%CI: [0.46–0.74]) and brain age gap (random effects model: *β* = 0.55, *p* < 0.001, 95%CI: [0.26–0.87]). But whereas there was little heterogeneity across DunedinPACNI effects (*I*^2^ = 12.6%, *p* = 0.32), there was substantial heterogeneity across brain age gap effects (*I*^2^ = 85.9%, *p* < 0.001). This heterogeneity should be interpreted with caution due to the limited number of datasets included in the meta-analysis. Full meta-analytic results are presented in Supplementary Figure S5.

## Discussion

Across four datasets, our analyses revealed evidence of accelerated aging in adults with schizophrenia as indexed by DunedinPACNI. We did not find evidence for accelerated aging in youth at clinical high-risk for psychosis. Patients with schizophrenia had faster DunedinPACNI compared to their unaffected siblings, who had similar DunedinPACNI scores as healthy controls. We did not identify confounding effects from tobacco smoking or antipsychotic medication use. The association between schizophrenia and faster DunedinPACNI was larger in comparisons between the oldest cases and the oldest controls. Finally, we observed that schizophrenia was associated with DunedinPACNI over and above the association with brain age gap. Collectively, these results are further evidence that schizophrenia is associated with accelerated aging beyond shared familial risk, smoking, or iatrogenic medication effects. Furthermore, our results suggest that this pattern may not become apparent until after conversion to psychosis and that this pattern is more pronounced among older schizophrenia patients. These results align with prior evidence of faster DunedinPACE—the epigenetic parallel of DunedinPACNI—across five case–control studies of schizophrenia (Caspi et al., 2024). The present results build on prior reports that have studied the brain age gap in schizophrenia (Constantinides et al., 2023; Kaufmann et al., 2019; Tønnesen et al., 2020) by showing that schizophrenia patients’ brains not only look chronologically older but also that patients with schizophrenia may experience accelerated ongoing aging.

The exact mechanism of accelerated aging in schizophrenia remains unclear and cannot be determined from our findings alone. Indeed, it likely occurs through multiple simultaneous and non-mutually exclusive causal pathways. One possible pathway is that accelerated aging occurs as an inherent component of schizophrenia. This possibility is supported by evidence that polygenic risk for schizophrenia is correlated with risk for a large number of non-psychiatric illnesses (Zhang et al., 2022). However, it is worth noting that prior research has not found evidence of genetic correlation between schizophrenia and older looking brains (Jawinski et al., 2025; Leonardsen et al., 2023). Testing for a genetic link between schizophrenia and accelerated aging awaits more comprehensive analyses of the genetic architecture of pace of aging measures, including DunedinPACNI and DunedinPACE. Another explanation is that certain behaviors typical of schizophrenia may, in turn, cause faster aging. For example, patients with schizophrenia are more likely to smoke tobacco, have a poor diet, and experience poverty, social isolation, and stress (Beam et al., 2024; de Leon & Diaz, 2005; Eglit et al., 2018; Fazel, Khosla, Doll, & Geddes, 2008; Folsom et al., 2005; Freilich et al., 2024; Gayer-Anderson & Morgan, 2013; Klopack et al., 2022; Maunakea et al., 2024; Mueser et al., 1990; Nannini et al., 2022; Volkow, 2009). Tobacco smoking in particular is well-known to be correlated with late-life disease risk (Kenfield et al., 2010; Prescott et al., 1998) and is correlated with accelerated aging (Klopack et al., 2022), making it plausible that high rates of smoking in schizophrenia contribute to faster aging. However, our current findings with DunedinPACNI, and prior findings with DunedinPACE, are robust to tobacco smoking, though with attenuated effects. Thus, tobacco smoking may explain some, but not all, of the association between schizophrenia and accelerated aging. A third explanation is that schizophrenia and accelerated aging share underlying causes; e.g. early-life adversity and long-term cannabis use contribute to both schizophrenia risk (Baldwin et al., 2023; Marconi et al., 2016) and accelerated aging (Belsky et al., 2022; Nannini et al., 2022; Yusupov et al., 2023). Similarly, there is overlap in genetic risk for schizophrenia and several age-related diseases (Chen et al., 2022; Hackinger et al., 2018; Pillinger et al., 2023), suggesting that common genes may confer risk for both schizophrenia and accelerated aging. Although our analyses of unaffected siblings suggest that shared familial risk factors are not driving both schizophrenia and accelerated aging, siblings still differ in early-life adversity, cannabis use, and genetic makeup. Therefore, our analyses of unaffected siblings do not rule out that these factors may confer risk for both schizophrenia and accelerated aging.

Although antipsychotic medication exposure was not associated with faster aging in our current analyses or prior analyses with DunedinPACE (Caspi et al., 2024), we cannot rule out that antipsychotic exposure contributes to accelerated aging. Antipsychotic medications are associated with adverse side effects on physical health (Hu et al., 2025; Jonas et al., 2021; Taipale et al., 2020). Further, our sample size with antipsychotic data was reduced (Supplementary Material S2), limiting our ability to detect small effects. However, antipsychotic use in schizophrenia also has benefits. For example, antipsychotic use in schizophrenia is associated with reduced psychotic relapse, lower risk of hospitalization due to physical illness, and lower all-cause mortality (Taipale et al., 2020), and experimental evidence suggests that antipsychotic treatment has protective effects on brain structure in first episode psychosis (Chopra et al., 2025). In addition, a recent study found no evidence for a dose-dependent relationship between antipsychotic use and epigenetic aging (Nader et al., 2025). The convergence between imaging and epigenetic aging measures also helps rule out that accelerated epigenetic aging in schizophrenia is simply due to antipsychotic effects on white blood cell counts (Siskind et al., 2025). Finally, early mortality in schizophrenia was also historically reported before the introduction of antipsychotics (Kirkpatrick et al., 2008), indicating an association between schizophrenia and poor health existed in the absence of antipsychotics. Untangling the various possible influences of antipsychotics on aging will require longitudinal, dose-variant, and genetically informed studies.

Our study has limitations. First, the participants in this analysis are younger than the participants of the Dunedin Study, which is the training dataset of DunedinPACNI. While DunedinPACNI detects disease risk among adults ranging from midlife to old age (Whitman et al., 2025), it is not yet known if there is reduced accuracy of DunedinPACNI in younger people. Future analyses using large normative samples may help establish whether there is any reduced validity of DunedinPACNI among young adults. Of note, all our analyses controlled for age, and sensitivity analyses using age-matched control groups in UNIBA-1 and UNIBA-2 yielded similar findings, so the observed case–control differences in DunedinPACNI are not explained by the chronological age of the samples. Relatedly, we cannot guarantee that our results will generalize to schizophrenia patients of advanced age. However, our strategy of studying young and middle-aged adults reduces the impact of survival bias in studies of older patients given earlier mortality in schizophrenia (Hjorthøj et al., 2017). Second, we cannot establish a causal link between schizophrenia and accelerated aging. Future research that uses quasi-experimental or clinical trial designs will be better suited to explore causation. Third, many youth at clinical high-risk for psychosis will not convert to a psychotic disorder; therefore, we can only hypothesize about when accelerated aging becomes apparent in the disease course of schizophrenia. Future longitudinal studies will be able to test whether there is a point in the psychosis disease progression where aging begins to accelerate. Fourth, common aging biomarkers, including DunedinPACNI, do not directly measure hallmarks of cellular aging (López-Otín et al., 2023). Therefore, we can neither establish how closely DunedinPACNI scores reflect underlying cellular aging processes nor whether these cellular aging processes cause (or are caused by) the structural brain patterns which drive DunedinPACNI. Further research linking aging biomarkers and structural brain MRI measures to cellular aging processes will help uncover how cellular hallmarks of aging may be altered in schizophrenia. Fifth, comprehensive data on health status and sociodemographic factors were not available, limiting our ability to draw mechanistic inferences. Furthermore, cigarette smoking and antipsychotic exposure were only available for a subset of participants, potentially reducing generalizability for these sensitivity tests.

Our findings add to a growing body of evidence supporting the hypothesis that accelerated aging is present in schizophrenia, which may contribute to the higher risk and earlier occurrence of age-related diseases. Future clinical trials should examine whether interventions targeting aging-related diseases (Bischoff-Ferrari et al., 2025; D’Ambrosio et al., 2025; van Dyck et al., 2023; Waziry et al., 2023; Weintraub et al., 2011) reduce morbidity among schizophrenia patients.

## Supporting information

10.1017/S003329172610333X.sm001 Whitman et al. supplementary material Whitman et al. supplementary material

## Supplementary material

The supplementary material for this article can be found at http://doi.org/10.1017/S003329172610333X.
