# Supplementary material for: Replicated evidence for an accelerated rate of whole-body aging in schizophrenia
Source: Psychol Med. 2026 Feb 9;56:e42. doi: 10.1017/S003329172610333X (PMC12902439; doi:10.1017/S003329172610333X)
Supplement: Whitman et al. supplementary material [file S003329172610333Xsup001.docx]

Supplemental Materials for:

**Replicated evidence for an accelerated rate of whole-body aging in schizophrenia**

Ethan T. Whitman et al.

Table of Contents

[Supplemental Material S1. Testing whether tobacco smoking accounted for accelerated aging in patients with schizophrenia vs. controls. 3](#_Toc219105938)

[Supplemental Material S2. Testing whether antipsychotic medication accounted for accelerated aging in patients with schizophrenia vs. controls. 5](#_Toc219105939)

[Supplemental Table S1. Group differences in DunedinPACNI and brain age gap in four datasets: LIBD, UNIBA-1, UNIBA-2, and NAPLS-3. 6](#_Toc219105940)

[Supplemental Table S2. Age-stratified analyses of schizophrenia-healthy control differences in LIBD, UNIBA-1, and UNIBA-2. 7](#_Toc219105941)

[Supplemental Table S3. Independent and combined associations between diagnosis, DunedinPACNI, and brain age gap in LIBD, UNIBA-1, UNIBA-2, and NAPLS-3. 8](#_Toc219105942)

[Supplemental Figure S1. Age distributions of cases and controls in LIBD, UNIBA-1, UNIBA-2, and NAPLS-3. 9](#_Toc219105943)

[Supplemental Figure S2. Association between cigarette smoking and DunedinPACNI in LIBD, UNIBA-1, and UNIBA-2. 10](#_Toc219105944)

[Supplemental Figure S3. Association between antipsychotic medication and DunedinPACNI in LIBD, UNIBA-1, and UNIBA-2. 11](#_Toc219105945)

[Supplemental Figure S4. Age-stratified schizophrenia-healthy control differences in DunedinPACNI in LIBD, UNIBA-1, and UNIBA-2. 12](#_Toc219105946)

[Supplemental Figure S5. Meta analyses of associations between schizophrenia, DunedinPACNI, and brain age gap. 13](#_Toc219105947)

# **Supplemental Material S1. Testing whether tobacco smoking accounted for accelerated aging in patients with schizophrenia vs. controls.**

We conducted sensitivity analyses to test whether tobacco smoking accounted for accelerated aging in patients with schizophrenia compared to controls. Of the 1,435 participants, 745 had available smoking data.

**Sample with available smoking data in LIBD, UNIBA-1, and UNIBA-2.**

|  | **Full sample** | | | | **Smoking available** | | | |
| --- | --- | --- | --- | --- | --- | --- | --- | --- |
| **Dataset** | **Total *N*** | **SCZ *N*** | **SIB *N*** | **HC *N*** | **Total *N*** | **SCZ *N*** | **SIB *N*** | **HC *N*** |
| LIBD | 416 | 112 | 46 | 258 | 301 | 72 | 24 | 205 |
| UNIBA-1 | 694 | 113 | 59 | 522 | 323 | 15 | 8 | 300 |
| UNIBA-2 | 325 | 66 | 11 | 248 | 121 | 27 | 0 | 94 |
| Abbreviations: HC = healthy control, LIBD = Lieber Institute for Brain Development, SCZ = schizophrenia, SIB = unaffected sibling, UNIBA = University of Bari. | | | | | | | | |

Although participants with available smoking data were slightly younger than those without (B=-1.54, *p*=0.002, 95% CI: [-2.51--0.57]), there were no significant differences in DunedinPACNI between participants with and without smoking data. We observed no sex differences in availability of smoking data (χ^2^ = 1.1, p = 0.29). We observed some difference in diagnosis prevalence (χ^2^ = 63.0, p < 0.001), with healthy controls appearing relatively more likely to have available smoking data.


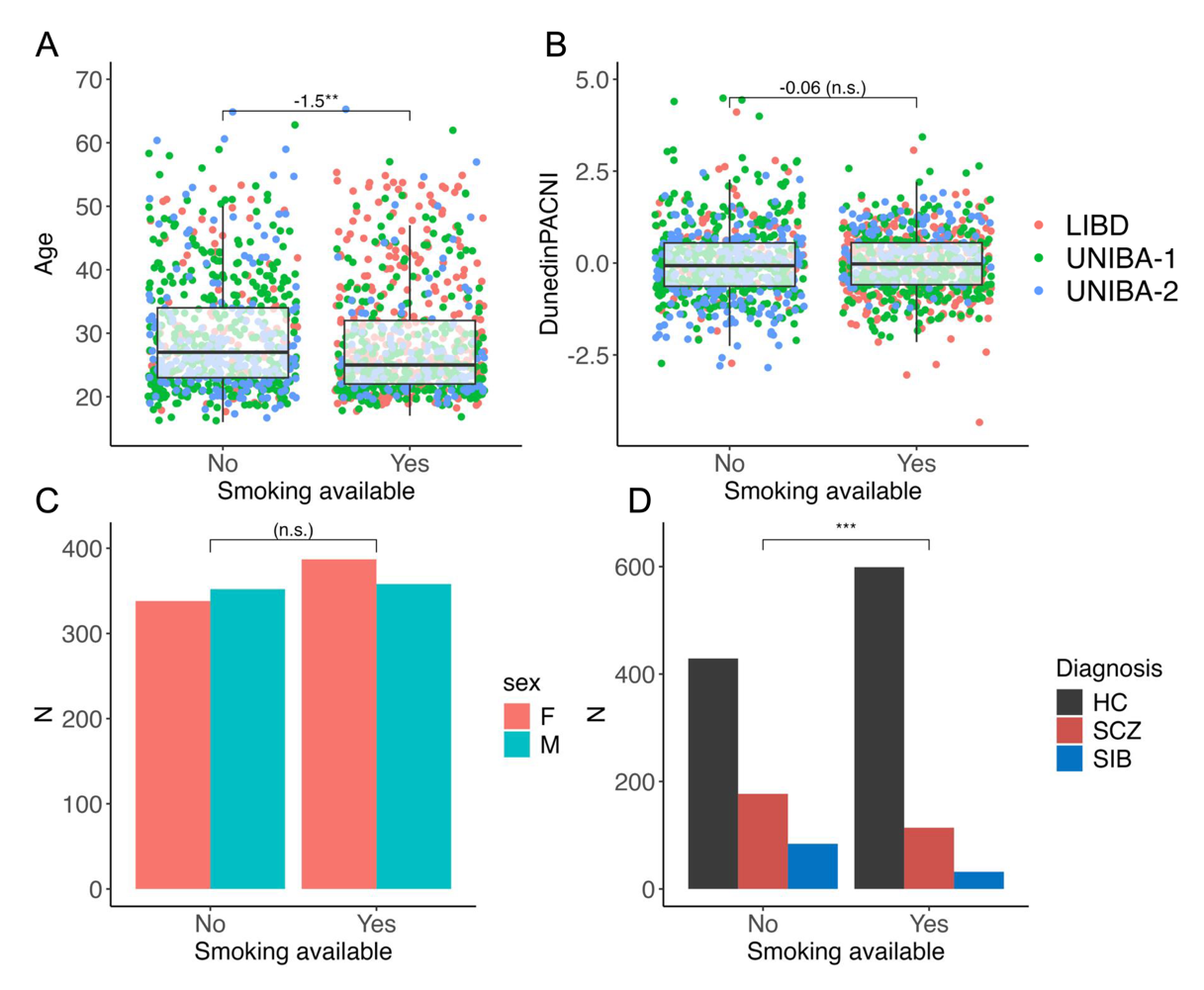


**Differences in age and DunedinPACNI between participants with and without available smoking data in LIBD, UNIBA-1, and UNIBA-2.** Boxplots showing group differences in **A.** age (unstandardized), **B.** DunedinPACNI (standardized), **C.** sex, and **D.** diagnosis among participants with and without available smoking data in LIBD, UNIBA-1, and UNIBA-2. Brackets in **A** and **B** represent standardized group differences. Brackets in **C** and **D** represent significance of chi square tests. **p < 0.01, ***p < 0.001. Abbreviations: F = female, HC = healthy control, LIBD = Lieber Institute for Brain Development, M = male, n.s. = not statistically significant, SCZ = schizophrenia, SIB = sibling, UNIBA = University of Bari.

Given the smaller available number of participants with smoking data (N=745), we pooled data across LIBD, UNIBA-1, and UNIBA-2 to perform a mega-analysis while covarying for dataset. As reported in the Results of the Main Article, we observed faster DunedinPACNI in schizophrenia compared to healthy controls while covarying for smoking (*β*=0.32, *p*=0.001, 95% CI: [0.13-0.51]).

# **Supplemental Material S2. Testing whether antipsychotic medication accounted for accelerated aging in patients with schizophrenia vs. controls.**

We conducted sensitivity analyses to test whether tobacco smoking accounted for accelerated aging in patients with schizophrenia compared to controls. Information about antipsychotic medication was available for 198 schizophrenia patients out of 291 patients.

**Sample with available antipsychotic data in LIBD, UNIBA-1, and UNIBA-2.**

| **Dataset** | **Total SCZ *N*** | **SCZ with chlorpromazine *N*** |
| --- | --- | --- |
| LIBD | 112 | 73 |
| UNIBA-1 | 113 | 77 |
| UNIBA-2 | 66 | 48 |
| Abbreviations: LIBD = Lieber Institute for Brain Development, SCZ = schizophrenia, UNIBA = University of Bari. | | |

There were no significant differences in chronological age or in DunedinPACNI between patients for whom there is or is not medication information. There were sex differences in availability of medication information (χ^2^ = 4.5, p = 0.03), with male patients being slightly more likely to be missing medication data.


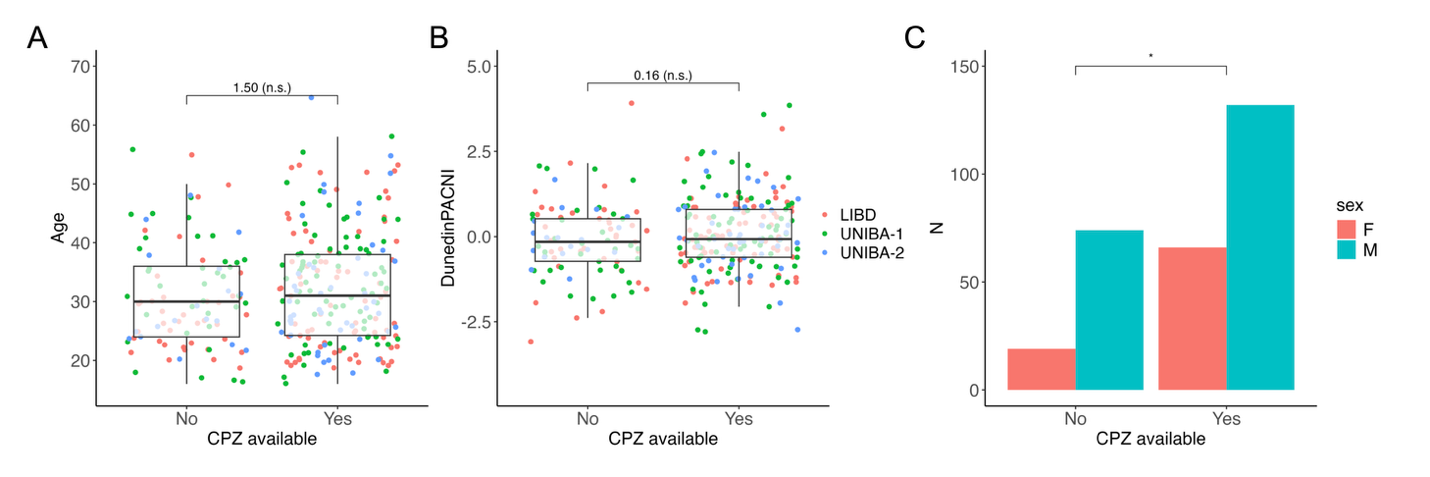


**Differences in age and DunedinPACNI between patients with and without available antipsychotic data in LIBD, UNIBA-1, and UNIBA-2.** Boxplots showing group differences in **A.** age (unstandardized) and **B.** DunedinPACNI (standardized), and **C.** sex among participants with and without available antipsychotic medication data in LIBD, UNIBA-1, and UNIBA-2. Brackets in **A** and **B** represent standardized group differences. Brackets in **C** represent significance of a chi square test. *p < 0.05. Abbreviations: CPZ = chlorpromazine, F = female, LIBD = Lieber Institute for Brain Development, M = male, n.s. = not statistically significant, UNIBA = University of Bari.

Given the smaller available number of participants with medication data (N=198), we pooled data across LIBD, UNIBA-1, and UNIBA-2 to perform a mega-analysis while covarying for dataset. As reported in the Results of the Main Article, we observed faster DunedinPACNI in schizophrenia compared to healthy controls while covarying for total chlorpromazine equivalents (*β*=0.44, *p*=0.002, 95% CI: [0.16-0.71]).

# **Supplemental Table S1. Group differences in DunedinPACNI and brain age gap in four datasets:** **LIBD, UNIBA-1, UNIBA-2, and NAPLS-3.**

| **Comparisons** | **Dataset** | **Measure** | ***N*** | **beta** | ***p*** | **95% CI** |
| --- | --- | --- | --- | --- | --- | --- |
| Case vs. HC | LIBD | DunedinPACNI | 370 | 0.48 | < .001*** | [0.27, 0.69] |
|  | LIBD | Brain age gap | 370 | 0.34 | < .001*** | [0.16, 0.51] |
|  | UNIBA-1 | DunedinPACNI | 635 | 0.70 | < .001*** | [0.50, 0.90] |
|  | UNIBA-1 | Brain age gap | 635 | 0.85 | < .001*** | [0.65, 1.05] |
|  | UNIBA-2 | DunedinPACNI | 314 | 0.62 | < .001*** | [0.36, 0.89] |
|  | UNIBA-2 | Brain age gap | 314 | 0.51 | < .001*** | [0.24, 0.78] |
|  | NAPLS-3 | DunedinPACNI | 659 | 0.12 | . 294 | [-0.10, 0.33] |
|  | NAPLS-3 | Brain age gap | 659 | 0.06 | . 581 | [-0.15, 0.26] |
| Case vs. SIB | LIBD | DunedinPACNI | 304 | 0.51 | .001** | [0.20, 0.83] |
|  | LIBD | Brain age gap | 304 | 0.22 | .112 | [-0.05, 0.48] |
|  | UNIBA-1 | DunedinPACNI | 581 | 0.64 | < .001*** | [0.34, 0.94] |
|  | UNIBA-1 | Brain age gap | 581 | 0.98 | < .001*** | [0.68, 1.28] |
|  | UNIBA-2 | DunedinPACNI | 259 | 0.19 | .535 | [-0.42, 0.81] |
|  | UNIBA-2 | Brain age gap | 259 | 0.60 | .063 | [-0.03, 1.23] |
|  | NAPLS-3 | DunedinPACNI | - | - | - | - |
|  | NAPLS-3 | Brain age gap | - | - | - | - |
| SIB vs. HC | LIBD | DunedinPACNI | 158 | -0.03 | .818 | [-0.32, 0.25] |
|  | LIBD | Brain age gap | 158 | 0.12 | .324 | [-0.12, 0.36] |
|  | UNIBA-1 | DunedinPACNI | 172 | 0.06 | .673 | [-0.21, 0.32] |
|  | UNIBA-1 | Brain age gap | 172 | -0.13 | .333 | [-0.40, 0.13] |
|  | UNIBA-2 | DunedinPACNI | 77 | 0.43 | .157 | [-0.16, 1.02] |
|  | UNIBA-2 | Brain age gap | 77 | -0.09 | .774 | [-0.70, 0.52] |
|  | NAPLS-3 | DunedinPACNI | - | - | - | - |
|  | NAPLS-3 | Brain age gap | - | - | - | - |
| Abbreviations: HC = healthy control, LIBD = Lieber Institute for Brain Development, NAPLS-3 = North American Prodrome Longitudinal Study - 3, SIB = sibling, UNIBA = University of Bari.  *Note:* Case refers to the schizophrenia group for LIBD, UNIBA-1, and UNIBA-2 and the clinical high-risk group for NAPLS-3 | | | | | | |

# **Supplemental Table S2. Age-stratified analyses of schizophrenia-healthy control differences in LIBD, UNIBA-1, and UNIBA-2.**

| **Dataset** | | **Age tertile** | ***N*** | ***N* SCZ** | ***N* HC** | **Mean age** | **SD age** | **Age range** | **beta** | ***p*** | **95% CI** |
| --- | --- | --- | --- | --- | --- | --- | --- | --- | --- | --- | --- |
| LIBD | | Young | 124 | 42 | 82 | 22.16 | 1.94 | 18-25 | 0.35 | .050 | [0.70, 0.00] |
|  |  | Middle | 123 | 31 | 92 | 27.98 | 2.15 | 25-32 | 0.50 | .014* | [0.89, 0.11] |
|  |  | Old | 123 | 39 | 84 | 42.49 | 6.95 | 32-55 | 0.50 | .005** | [0.84, 0.16] |
| UNIBA-1 | | Young | 212 | 14 | 198 | 20.75 | 1.27 | 16-23 | 0.61 | .016* | [1.10, 0.12] |
|  |  | Middle | 212 | 19 | 193 | 25.19 | 1.60 | 23-28 | 0.62 | .002** | [1.00, 0.23] |
|  |  | Old | 211 | 80 | 131 | 37.46 | 7.38 | 28-63 | 0.73 | < .001*** | [1.03, 0.43] |
| UNIBA-2 | | Young | 105 | 11 | 94 | 21.33 | 1.43 | 17-24 | 0.14 | .633 | [0.73, -0.45] |
|  |  | Middle | 105 | 20 | 85 | 25.70 | 1.24 | 24-28 | 0.68 | .006** | [1.16, 0.20] |
|  |  | Old | 104 | 35 | 69 | 37.25 | 9.18 | 28-65 | 0.86 | < .001*** | [1.23, 0.50] |
|  |  |  |  |  |  |  |  |  |  |  |  |

Abbreviations: CI = confidence interval, HC = healthy control, LIBD = Lieber Institute for Brain Development, Max. = maximum, Min. = minimum, SD = standard deviation, UNIBA = University of Bari.

# **Supplemental Table S3. Independent and combined associations between diagnosis, DunedinPACNI, and brain age gap in LIBD, UNIBA-1, UNIBA-2, and NAPLS-3.**

|  |  | | | **Independent models** | | | | **Combined models** | | |
| --- | --- | --- | --- | --- | --- | --- | --- | --- | --- | --- |
| **Dataset** | **Measure** | | | **beta** | | ***p*** | **95% CI** | **beta** | ***p*** | **95% CI** |
| LIBD | DunedinPACNI | | | 0.48 | | < .001*** | [0.27, 0.69] | 0.44 | < .001*** | [0.23, 0.65] |
| LIBD | Brain age gap | | | 0.34 | | < .001*** | [0.16, 0.51] | 0.30 | .001** | [0.12, 0.48] |
| UNIBA-1 | DunedinPACNI | | | 0.70 | | < .001*** | [0.50, 0.90] | 0.51 | < .001*** | [0.30, 0.71] |
| UNIBA-1 | Brain age gap | | | 0.85 | | < .001*** | [0.65, 1.05] | 0.69 | < .001*** | [0.49, 0.89] |
| UNIBA-2 | DunedinPACNI | | | 0.62 | | < .001*** | [0.36, 0.89] | 0.60 | < .001*** | [0.33, 0.87] |
| UNIBA-2 | Brain age gap | | | 0.51 | | < .001*** | [0.24, 0.78] | 0.49 | .001*** | [0.21, 0.77] |
| NAPLS-3 | DunedinPACNI | | | 0.12 | | .294 | [-0.10, 0.33] | 0.10 | .335 | [-0.11, 0.31] |
| NAPLS-3 | Brain age gap | | | 0.06 | | .581 | [-0.15, 0.26] | 0.04 | .688 | [-0.16, 0.24] |
|  | |  |  | |  |  |  |  |  |  |

Abbreviations: HC = healthy control, LIBD = Lieber Institute for Brain Development, NAPLS-3 = North American Prodrome Longitudinal Study - 3, SIB = sibling, UNIBA = University of Bari.

*Note:* Diagnosis refers to schizophrenia for LIBD, UNIBA-1, and UNIBA-2 and clinical high-risk status for NAPLS-3.

*Note:* Combined models include both DunedinPACNI and brain age gap.


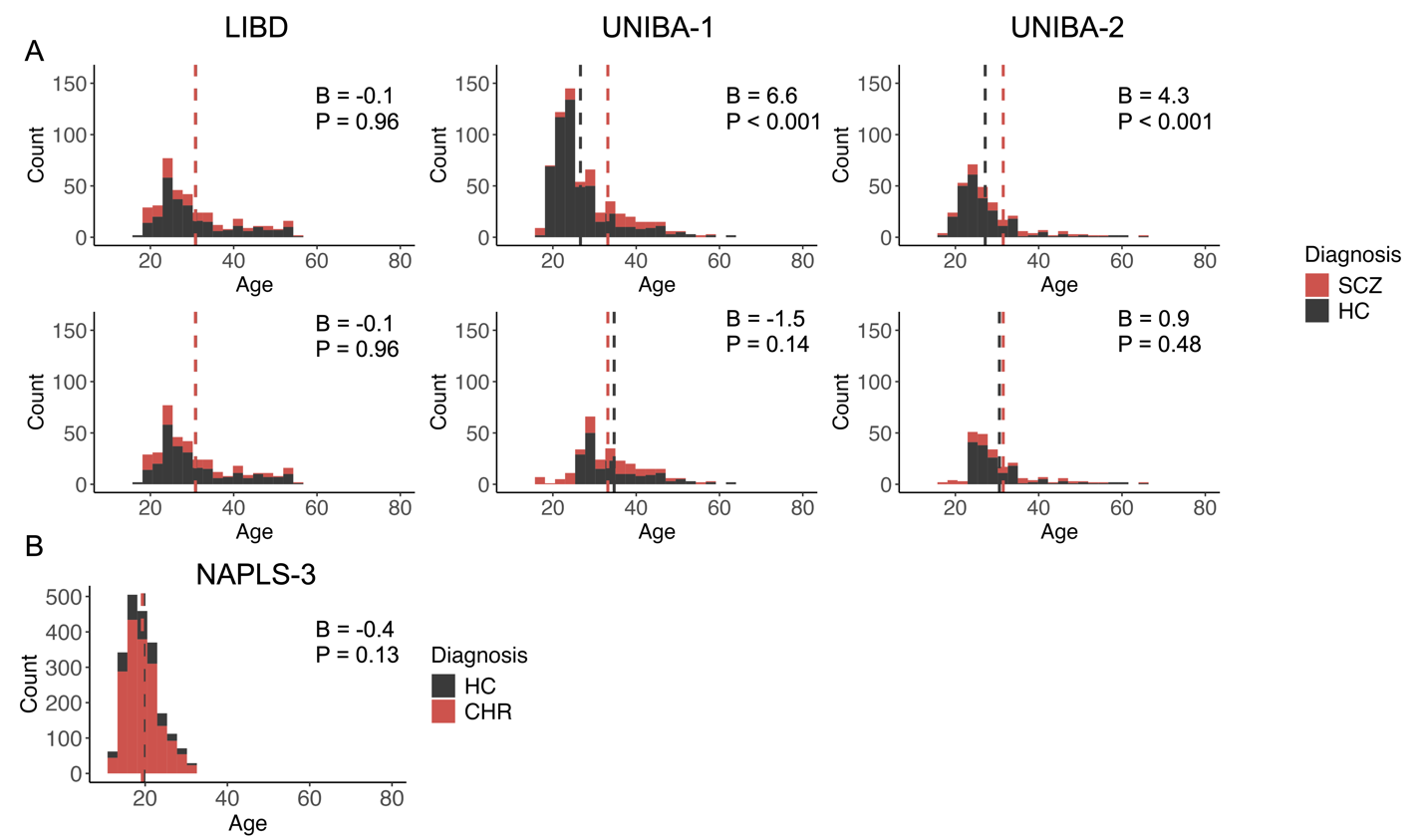


**Supplemental Figure S1. Age distributions of cases and controls in LIBD, UNIBA-1, UNIBA-2, and NAPLS-3. A.** Histograms showing age distributions in LIBD, UNIBA-1, and UNIBA-2. Effect sizes of mean difference in years between schizophrenia patients and controls are overlaid on each plot. Top row shows age distributions for full datasets, bottom row shows age distributions in age-matched control sensitivity analyses. Note that there is no change in the bottom panel of LIBD age distributions, as these groups do not differ by chronological age in the overall sample. Vertical lines show the mean age of each group. Red indicates schizophrenia patients, black indicates healthy controls. **B.** Histogram showing age distribution in NAPLS-3. Effect size of mean difference in years between clinical high risk and healthy control participants is overlaid. Vertical lines show mean age of each group. Red indicates clinical high risk, black indicates healthy control. Abbreviations: CHR = clinical high risk for psychosis, HC = healthy control, LIBD = Lieber Institute for Brain Development, SCZ = schizophrenia, UNIBA = University of Bari.


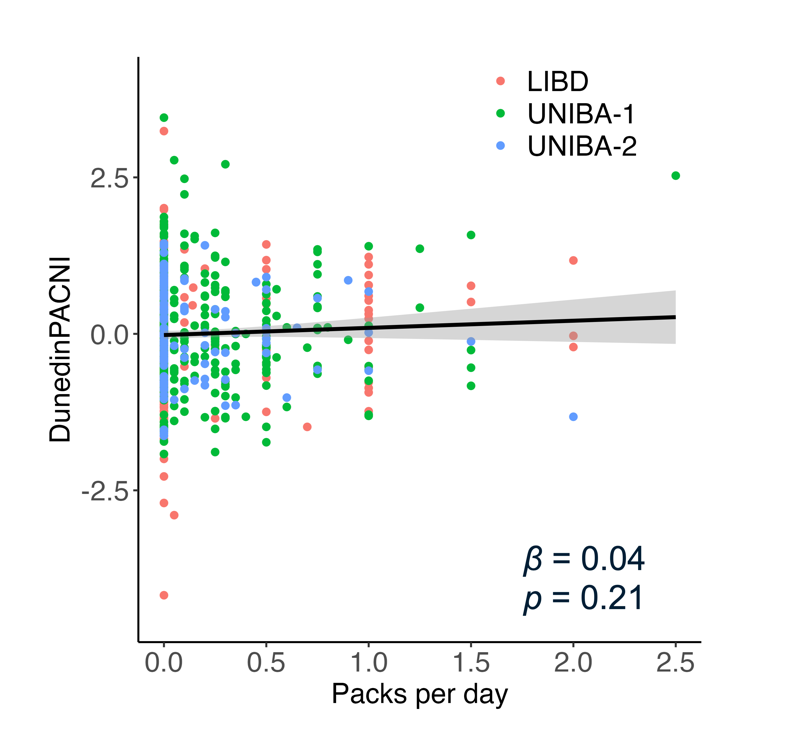


**Supplemental Figure S2. Association between cigarette smoking and DunedinPACNI in LIBD, UNIBA-1, and UNIBA-2.** Scatterplot showing linear associations between current packs smoked per day and DunedinPACNI scores in LIBD, UNIBA-1, and UNIBA-2. Abbreviations: LIBD = Lieber Institute for Brain Development, UNIBA = University of Bari.

**
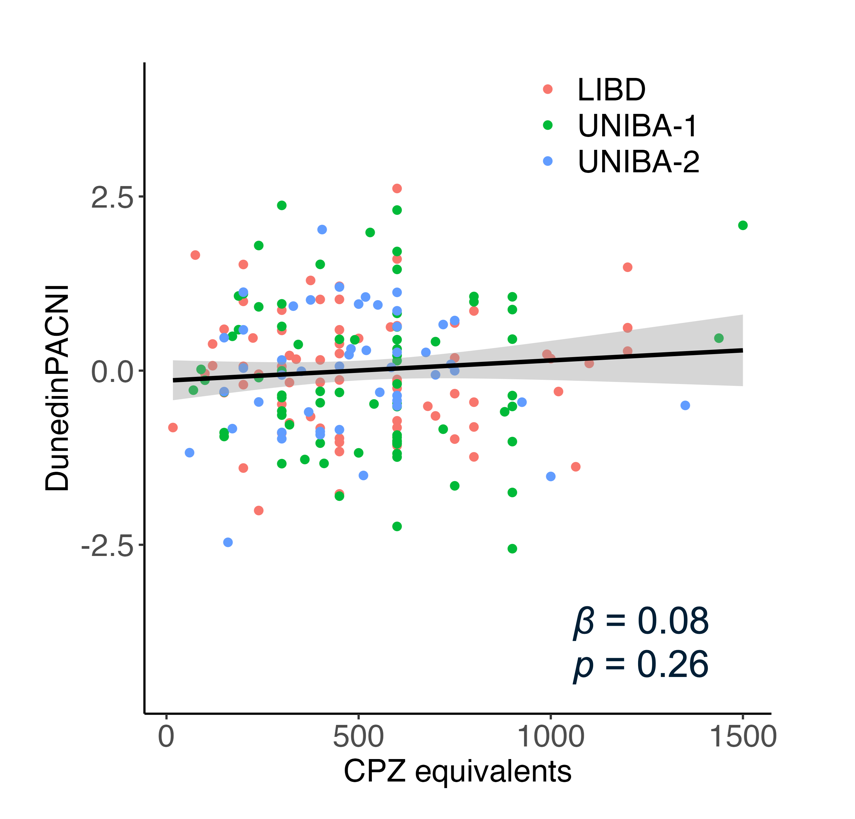
**

**Supplemental Figure S3. Association between antipsychotic medication and DunedinPACNI in LIBD, UNIBA-1, and UNIBA-2.** Scatterplot showing linear associations between lifetime chlorpromazine exposure and DunedinPACNI scores in LIBD, UNIBA-1, and UNIBA-2. Abbreviations: CPZ = chlorpromazine, LIBD = Lieber Institute for Brain Development, SCZ = schizophrenia, UNIBA = University of Bari.


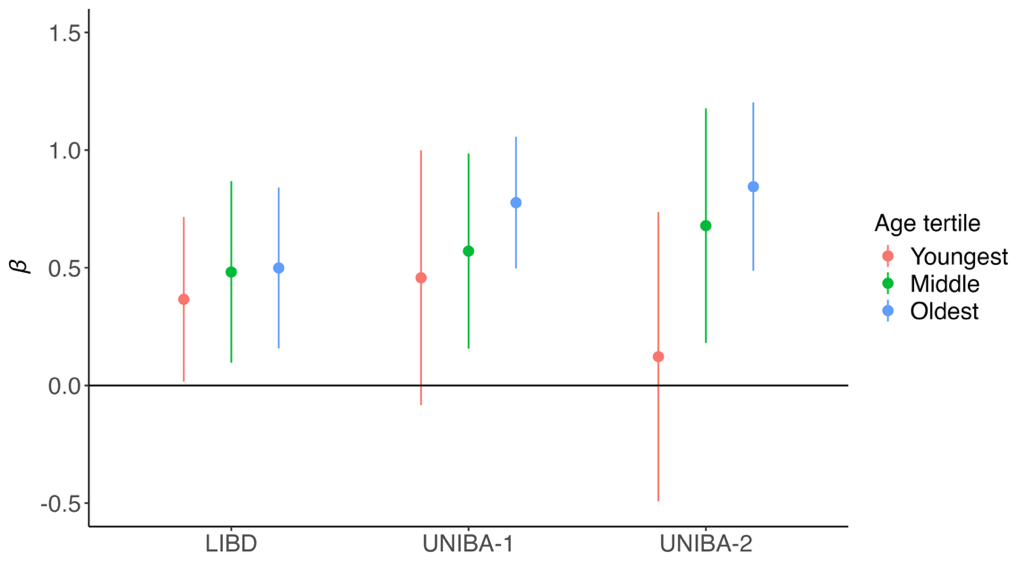


**Supplemental Figure S4. Age-stratified schizophrenia-healthy control differences in DunedinPACNI in LIBD, UNIBA-1, and UNIBA-2.** Forest plot of standardized mean differences between schizophrenia patients and healthy controls when grouping participants into age tertiles. Age tertiles were calculated within each dataset using only schizophrenia patients and healthy controls. Demographic details for each age subset are presented in **Supplemental Table S2**. All analyses control for age and sex. Error bars represent 95% confidence intervals. Abbreviations: LIBD = Lieber Institute for Brain Development; UNIBA = University of Bari.


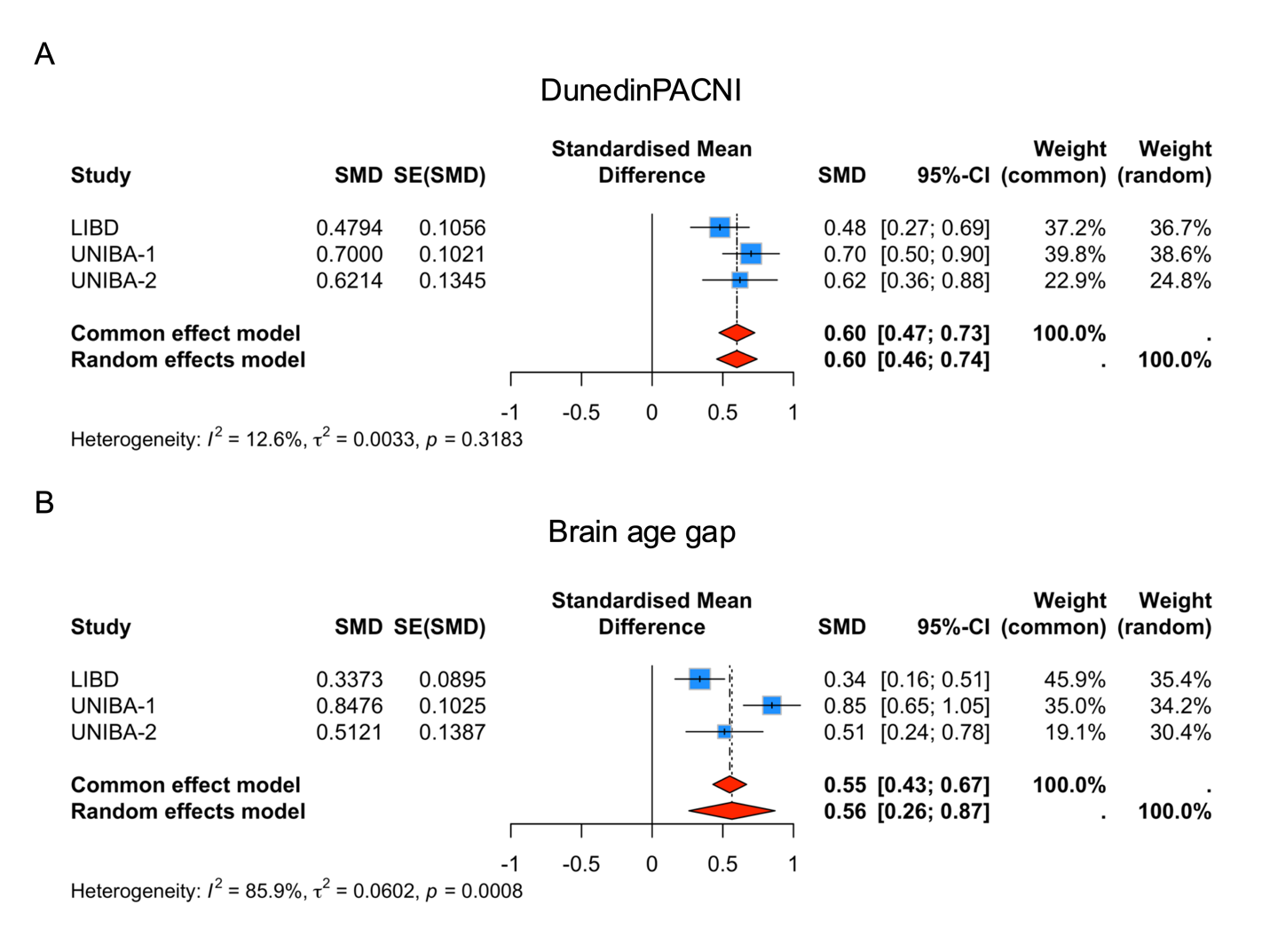


**Supplemental Figure S5. Meta analyses of associations between schizophrenia, DunedinPACNI, and brain age gap.** Forest plots of standardized mean differences between schizophrenia and control groups in **A.** DunedinPACNI and **B**. brain age gap. Error bars represent 95% confidence intervals. All analyses control for age and sex. Abbreviations: CI = confidence interval, LIBD = Lieber Institute for Brain Development, SE = standard error, SMD = standardized mean difference, UNIBA = University of Bari
